# Family Socioeconomic Status and Adolescent School Satisfaction: Does Schoolwork Support Affect This Association?

**DOI:** 10.3389/fpsyg.2022.841499

**Published:** 2022-03-30

**Authors:** Simona Horanicova, Daniela Husarova, Andrea Madarasova Geckova, Andrea F. de Winter, Sijmen A. Reijneveld

**Affiliations:** ^1^Department of Health Psychology and Methodology Research, Faculty of Medicine, Pavol Jozef Šafárik University in Košice, Košice, Slovakia; ^2^Department of Community and Occupational Health, University Medical Center Groningen, University of Groningen, Groningen, Netherlands; ^3^Institute of Applied Psychology, Faculty of Social and Economic Sciences, Comenius University in Bratislava, Bratislava, Slovakia

**Keywords:** adolescents, socioeconomic status, school satisfaction, schoolwork support, school

## Abstract

**Background:**

The aim of this study is to explore the association of family socioeconomic status (SES) and internal and external schoolwork support with adolescents’ school satisfaction and whether schoolwork support modifies these associations.

**Methods:**

Data come from the cross-sectional Health Behavior in School-aged Children study collected in 2018 from Slovak 15-year-olds (*N* = 1127; 52.7% boys). SES was measured by Family Affluence Scale (low; middle; high). School satisfaction was measured *via* school engagement and attitudes toward education. Schoolwork support was measured regarding two groups of sources inside and outside the family, separately. Logistic regression models were used to explore the associations of SES and schoolwork support with school satisfaction as well as the moderating effect of schoolwork support.

**Results:**

Adolescents with low SES were more likely to feel indifferent toward school and education (odds ratios/95%-confidence interval: 1.77/1.26–2.49), and similarly, adolescents who did not have schoolwork support inside or outside the family (1.38/1.02–1.87, and 1.50/1.01–2.22, respectively). Schoolwork support moderated the associations of SES with school satisfaction. Adolescents with low and middle SES without support inside or outside the family were more likely to feel indifferent than satisfied (2.72/1.21–6.10; 3.00/1.27–7.06; and 2.86/1.05–7.80; 6.04/1.72–21.24, respectively).

**Conclusion:**

Adolescents from low and middle SES without schoolwork support inside or outside the family are more likely to feel indifferent toward school and education.

## Introduction

Adolescents with disadvantaged backgrounds are more likely to have poor access to schooling and education, below-average performance and low motivation and persistence to stay at school compared to their more privileged peers (e.g., Wells, 2008; Fan, 2014; Kirkland et al., 2015; OECD, 2019). Parental social status and education levels impact their children’s academic trajectory, learning attitudes and access to educational resources (Fan, 2014).

### School Satisfaction

School satisfaction is one of the many terms used to describe adolescents’ attitudes toward school (Libbey, 2004). Satisfaction with school regards experienced mood, enthusiasm, happiness and boredom and the extent of pleasant experiences at school (Samdal et al., 1998; Kalil and Ziol-Guest, 2003). New approaches perceive school satisfaction as an important part of life satisfaction and quality of life (Baker and Maupin, 2009). Moreover, school satisfaction is considered a good indicator of the fulfillment of three main self-determination needs including autonomy, competence and relatedness (Ryan and Deci, 2017) through mastery-stimulating school environment and may later manifest in the areas of adolescents’ health and life satisfaction (Inchley et al., 2018). Lack of school satisfaction may thus affect adolescents’ mental and physical health (Ottova et al., 2012) and health risk behaviors (Vogel et al., 2015). Many adolescents in Slovakia report that they feel indifferent toward school and education; i.e., they don’t like school and they don’t care about their education at all (Bosakova et al., 2020; Horanicova et al., 2020), and this may also hold for other countries with similar educational system such as Czech Republic or Poland (Currie et al., 2012). Feeling indifferent toward school, in comparison with caring about education and liking school, occurs particularly among children who live in low-affluence families and a disrupted social context (Bosakova et al., 2020). The social background of adolescents may thus affect their attitudes and beliefs toward school and expectations for their future education (Ming Ming and Chow, 2015), which may result in a compromised academic and professional trajectory.

### Schoolwork Support

Many adolescents are able to overcome their socioeconomic challenges and achieve higher levels of academic proficiency through academic resilience (OECD, 2019), using several resources. More academically resilient adolescents are those who have a source of parental and teacher support (Martin and Marsh, 2006). Likewise, forming a strong relationship with a supportive adult was proven to be an important component of dealing with social inequalities and increasing the ability to learn among adolescents (Powell and Marshall, 2011). Moreover, support from teachers and classmates promotes adolescents’ satisfaction with school (Horanicova et al., 2020). Parental support with homework along with structured and amicable environment at home has been shown to increase adolescents’ motivation, positive emotions and self-efficacy toward school assignments (Madjar et al., 2016; Moè and Katz, 2018; Moè et al., 2018) also among families with lower-SES (O’Sullivan et al., 2014) and may therefore improve their performance at school. Likewise, autonomy-enhancing and friendly educators’ approach increases adolescents’ school engagement and motivation (Wang and Eccles, 2013). Adolescents may benefit from the support with schoolwork within their domestic environment through their parents. Many of these adolescents may, however, lack the much needed support from family due to their parental socioeconomic background, e.g., parents unable to dedicate sufficient time and resources due working multiple jobs (García and Weiss, 2018).

### The Role of Family Socioeconomic Status

Social inequalities are one of the most important non-cognitive factors that predict further academic outcomes of the adolescents and may modify their engagement and academic abilities (Wang and Eccles, 2013). Overcoming socioeconomic disadvantages is particularly important with regards to adolescents’ academic achievement and school engagement. Keeping adolescents engaged with school is of major importance for their academic success, grades and risk behaviors regarding e.g., juvenile delinquency, truancy, and drugs and alcohol abuse (Wang and Fredricks, 2014; Fredricks et al., 2016). Families differ with regards to the level of their social and cultural capital (Mills and Gale, 2007). Capitals are reproduced and inherited by further generations in the form of family educational views and values and may further affect cultural and educational environment and atmosphere for children (Fan, 2014). Although adolescents with disadvantaged backgrounds are more likely to experience poorer academic achievement and educational outcomes (Ferguson et al., 2007; Goodman et al., 2012), having a source of schoolwork support inside and/or outside of the family may help them prevent negative repercussions and improve their school satisfaction. Moreover, support with schoolwork from the inside and outside of the family may help to prevent the intergenerational transfer of socioeconomic disadvantages.

### Aim and Hypotheses

Previous research has mostly focused on the consequences of social inequalities on adolescents’ attitudes toward school and access to education (Wells, 2008; Fan, 2014; OECD, 2016). However, evidence on the associations of SES and school satisfaction and possible moderators that may reduce the undesirable consequences is scarce. We hypothesized that adolescents’ low SES would be associated with low school satisfaction, and that adolescents’ lack of schoolwork support would be associated with low school satisfaction. Additionally, we hypothesized that schoolwork support would moderate the association between SES and school satisfaction. Therefore, the aim of this study is to explore the association of family SES and internal and external schoolwork support with adolescents’ school satisfaction and whether schoolwork support modifies these associations.

## Materials and Methods

### Sample and Procedure

We used data from the Health Behavior in School-aged Children (HBSC) study conducted in 2018 in Slovakia as an online cross-sectional questionnaire-based survey on health and health-related behavior. The population-representative sample was obtained *via* two-step sampling. In the first step, 140 larger and smaller elementary schools from rural and urban areas from all regions of Slovakia were asked to participate. They were randomly selected from a list of all eligible schools in Slovakia obtained from the Slovak Institute of Information and Prognosis for Education. In the end, 109 schools agreed to participate in our survey (77.9%). In the second step, we obtained data from 8,405 adolescents from the fifth to ninth grades of these elementary schools, aged 11–15 years old (mean age 13.43; 50.9% boys). For the purpose of this study we only used data from 15-year-old adolescents (*N* = 1,293), who answered questions regarding their attitude toward education. Moreover, respondents with missing responses were excluded (*N* = 166), leading to a final sample of 1,127 adolescents.

The study was approved by the Ethics Committee of the Medical Faculty at P.J. Šafárik University in Košice (16N/2017). Parents were informed about the study *via* the school administration and could opt out if they disagreed with their child’s participation.

### Measures

*School satisfaction* regarded the combination of school engagement and attitudes toward education. *School engagement* was measured using the item: “How do you feel about school at present?,” with four-point Likert-type responses (“I like it a lot”; “I like it a bit”; “I don’t like it very much”; “I don’t like it at all”). *Attitudes toward education* were measured using the item “Do you care what kind of education you will have?,” with three-point Likert-type responses (“I care a lot”; “I care about it, but not too much”; “I could not care less”). Next, we created a binary variable of school satisfaction by creating two groups: (1) indifferent—adolescents who do not like school a lot and do not care about their education a lot, (2) others—adolescents who do not like school a lot but care about their education a lot, or adolescents who like school a lot but do not care a lot about their education, and those who like school a lot and care about their education a lot (Bosakova et al., 2020; Horanicova et al., 2020).

*Schoolwork support* was measured for two types of support: inside the family (*Internal;* mother or father; one of my grandparents; one of my siblings; someone else in my family) and outside the family (*External;* another adult outside my family; an adult at my school; one of my friends, peers). This was based on the question “Does anyone support you and help you with your schoolwork?,” with options including “Mother or father”; “One of my grandparents”; “One of my siblings”; “Someone else in the family”; “Another adult outside my family”; “An adult at my school”; “One of my friends, peers,” with three-point Likert-type responses for each option (Yes, regularly; Yes, sometimes; No). The answers were dichotomized to yes/no, with “yes” regarding “Yes” and “Yes regularly.” Next, we derived two groups of sources of help, internal and external, i.e., support within the family and outside the family, respectively. Responses for each group of source of help were then grouped into three categories: (1) those who didn’t have any source of support; (2) those who only had one source of support and (3) those who had two or more sources of support (Gecková, 2019).

*Family SES* was measured using the Family Affluence Scale III (FAS-III), which consists of six questions: “Does your family own a car, van or truck?” (No/Yes, one/Yes, two or more), “Do you have your own bedroom for yourself?” (Yes/No), “How many computers does your family own?” (None/One/Two/More than two), “How many bathrooms (room with a bath/shower or both) are in your home?” (None/One/Two/More than two), “Does your family have a dishwasher at home?” (Yes/No), “How many times did you and your family travel out of your country for a holiday/vacation last year?” (Not at all/Once/Twice/More than twice). The sum score was converted into a ridit score ranging from 0 to 1 with the mean (0.5) in the middle of the distribution. Next, we created three tertiles: low (0–0.333), medium (0.334–0.666) and high (0.667–1) socioeconomic position (Elgar et al., 2015).

### Statistical Analyses

Firstly, the baseline characteristics of the sample were described using descriptive statistics. Secondly, we assessed the associations of SES and sources of support, each separately, with school satisfaction in logistic regression analyses models adjusted for gender (Model 1). Thirdly, we entered SES and sources of support into the model adjusted for gender (Model 2). Finally, we added the interaction of SES and sources of support on school satisfaction to the model (Model 3). We followed this analytical strategy for internal and external sources of support separately. All analyses were performed using IBM SPSS Statistics 23 for Windows.

## Results

### Baseline Characteristics

Table 1 shows the characteristics of the sample. Up to 35% of the adolescents reported that they did not have any internal source of support, and almost a quarter of the adolescents reported having only one internal source of support. Almost 33% of the adolescents reported having no external source of support, and almost 46% of the adolescents reported having only one external source of support. Almost 30% of the adolescents reported that they were indifferent.

**TABLE 1:** Descriptive Statistics of the Sample (Slovakia 2018, 15-year-olds, *N* = 1127).

	*n* (in%)
**Gender**	
Boys	594 (52.7)
Girls	533 (47.3)
**Socioeconomic status**	
Low	324 (34.2)
Middle	275 (29.1)
High	347 (36.7)
**Internal sources of support**	
None	395 (36.5)
One	281 (25.9)
Two or more	408 (37.6)
**External sources of support**	
None	367 (33.9)
One	515 (47.6)
Two or more	201 (18.5)
**School satisfaction**	
Indifferent	328 (29.1)
Others	799 (70.9)

*Only valid percentages are presented, numbers of missing values regard: Socioeconomic status, 181, Internal sources of support, 43, External sources of support, 44.*

### Associations of Socioeconomic Status and Internal and External Sources of Support With School Satisfaction and Moderating Role of Schoolwork Support

**FIGURE 1:**
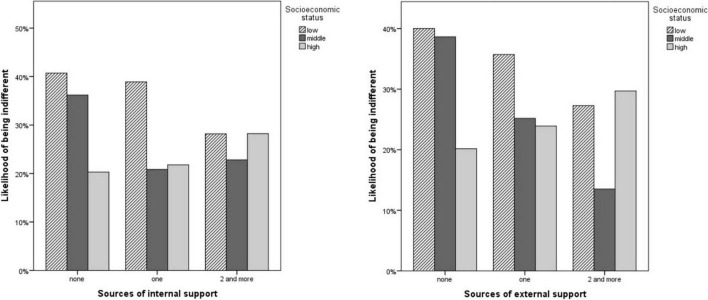
The likelihood of being indifferent while having none, one and two or more internal and external sources of support with regards to SES.

Adolescents who did not have any internal source of support and adolescents with low SES were more likely to be indifferent (Table 2). Likewise, adolescents with no external source of support and adolescents with low SES were more likely to be indifferent (Table 3). Figure 1 shows the likelihood of being indifferent to depend on the number of sources of internal and external support across the three levels of SES. The likelihood of feeling indifferent is lower among adolescents with a low SES who received support from one or more internal or external sources than for their peers who did not receive support.

**TABLE 2:** The associations of SES and the number of Internal sources of support with School satisfaction (Likelihood of Being Indifferent): Results from binomial logistic regression models adjusted for gender (Odds ratios, OR; 95% Confidence interval, CI) (Slovakia 2018, 15-year-olds, *N* = 1127).

	Model 1 (separate associations, adjusted for gender) OR (95% CI)	Model 2 (with mutual adjustment added) OR (95% CI)	Model 3 (with interaction added) OR (95% CI)
**SES**	**	**	
Low	1.77 (1.26–2.49)**	1.86 (1.32–2–63)***	1.00 (0.57–1.78)
Middle	1.29 (0.90–1.85)	1.28 (0.88–1.86)	0.79 (0.42–1.48)
High	1	1	1
**Source of support**	*		
None	1.38 (1.02–1.87)*	1.24 (0.88–1.73)	0.62 (0.35–1.11)
One	1.06 (0.75–1.50)	1.02 (0.70–1.50)	0.69 (0.35–1.35)
Two or more	1	1	1
SES*source of support			*
low SES*no source of support			2.72 (1.21–6.10)*
Middle SES*no source of support			3.00 (1.27–7.06)*
Low SES*one source of support			2.40 (0.98–5.89)
Middle SES*one source of support			1.22 (0.45–3.34)

*p < 0.05, **p < 0.01, ***p < 0.001.

*Model 1—associations of source of help and SES separately, adjusted for gender.*

*Model 2—associations of source of help and SES mutually adjusted, after adjustment for gender. Model*

*3—model with interaction of source of help and SES added.*

*1—Reference group.*

**TABLE 3:** The associations of SES and the number of external sources of support with school satisfaction (Likelihood of Being Indifferent): Results From Binomial Logistic Regression Models Adjusted for Gender (Odds ratios, OR; 95% Confidence interval, CI) (Slovakia 2018, 15-year-olds, *N* = 1,127).

	Model 1	Model 2	Model 3
	(Separate associations, adjusted for gender) OR (95% CI)	(With mutual adjustment added) OR (95% CI)	(With interaction added) OR (95% CI)
**SES**	**	**	
Low	1.77 (1.26–2.49)**	1.82 (1.29–2.57)**	0.93 (0.42–2.08)
Middle	1.29 (0.90–1.85)	1.29 (0.89–1.87)	0.41 (0.14–1.23)
High	1	1	1
**Source of support**	*		
None	1.50 (1.01–2.22)*	1.33 (0.85–2.07)	0.58 (0.29–1.18)
One	1.25 (0.86–1.83)	1.20 (0.78–1.82)	0.78 (0.41–1.49)
Two or more	1	1	1
SES*source of support			*
low SES*no source of support			2.86 (1.05–7.80)*
middle SES*no source of support			6.04 (1.72–21.24)**
Low SES*one source of support			1.94 (0.76–4.97)
Middle SES*one source of support			2.67 (0.80–8.95)

*p < 0.05, **p < 0.01.

*Model 1—associations of source of help and SES separately, adjusted for gender.*

*Model 2—associations of source of help and SES mutually adjusted, after adjustment for gender.*

*Model 3—model with interaction of source of help and SES added.*

*1—Reference group.*

Regarding the interactions with schoolwork support, adolescents with low and medium SES who did not have any internal nor external source of support were more likely to be indifferent (Tables 2, 3).

## Discussion

The aim of this study was to explore the associations of SES and schoolwork support inside and outside the family with school satisfaction and the degree to which the association of family SES and school satisfaction is modified by schoolwork support inside and outside the family. We found that adolescents with low SES and without schoolwork support were more likely to feel indifferent toward school and education. Moreover, schoolwork support moderated the associations of family SES and school satisfaction. Adolescents with low and middle SES without any source of internal or external support were more likely to be indifferent.

We found that the adolescents from low SES families were more likely to feel indifferent (i.e., don’t like school and don’t care about the education). These findings support previous research that emphasizes the connection between family socioeconomic background and adolescents’ perceptions of school and education (Evans et al., 2010; OECD, 2018, 2019). Socioeconomic characteristics of the family are naturally reproduced and passed onto children in the form of social and cultural capital through intergenerational inheritance (Bourdieu and Passeron, 1977; Fan, 2014). These acquired characteristics subsequently affect adolescent access to schooling, expectations for their future, which may include interest in further education, and their beliefs (OECD, 2019), including school satisfaction (Bosakova et al., 2020). A socially disadvantaged background of adolescents affects their attitudes toward school and education and may further impact their future academic and professional trajectory.

We also found that the adolescents who did not have any support within or outside the family were more likely to feel indifferent toward school and education. These results are in line with previous research showing the importance of assistance for adolescents who struggle at school (Quadlin, 2015). Parental involvement in children’s homework appears to be beneficial even after proceeding onto middle and high school (Xu and Corno, 2003). Providing adolescents with schoolwork help facilitates focusing on their individual values and improve the importance of education (Xu and Corno, 2003; Li and Hamlin, 2019). Without this kind of help, adolescents may find themselves struggling at school, and their attitudes toward school and education may deteriorate because of that struggle.

Additionally, we found that indifference toward school is particularly likely in the case of a lack of schoolwork support and low family SES. The likelihood of feeling indifferent toward school and education among low and middle SES adolescents appears to decrease with an increasing number of schoolwork support sources in or outside the family. These findings support previous evidence on the associations between socioeconomic inequities and educational resources and success (OECD, 2018, 2019; Thomson, 2018). The interpretation for these findings may lie in the attributes of less privileged children, who are more likely to attend an underperforming school and whose parents struggle with different working hours and may not have time to engage in their children’s schoolwork activities (García and Weiss, 2018). On top of that, resources to compensate for the lack of dedicated time may often be lacking due to the family financial situation. Involvement in adolescents’ schoolwork seems to be important in moderating the effect of the adolescents’ socioeconomic background and may help them prosper in further and academic journey and healthy development.

Surprisingly, the likelihood of being indifferent increased with the increasing amount of schoolwork support sources among adolescents with high SES. Although a lot of evidence points out that children with higher SES are more likely to be successful and perform better at school (e.g., Woods et al., 2005; OECD, 2016, 2018; García and Weiss, 2018), less attention has been paid to the struggles of growing up privileged. Privileged children may experience a lot of pressure from parents and teachers to deliver results (Becker and Luthar, 2002, 2007), which could affect their attitudes toward school and education; further exploration of these associations may be an interesting issue.

### Strengths and Limitations

The strengths of this study include its use of a large and representative sample of 15-year-old adolescents and following the international HBSC study protocols. Some limitations need to be mentioned, too. First, we used self-reported questionnaires, which may have caused some information bias, but we minimized this by using validated measures (Inchley et al., 2018). Second, we assessed schoolwork support using a single question. This did not allow us to differentiate between potentially less and more effective ways of schoolwork support. Next, the psychological characteristics of the children were not taken into an account, future research on this topic might take into account some of the psychological characteristics e.g., it is very likely that gifted children do not need school help even though they are from low SES. Additionally, we used adolescent-reported data on family SES. These may contain errors as not all adolescents know such information, making that we may have underestimated these associations; further research may complement data focused on parental job and educational level. Finally, the cross-sectional design of the study does not allow inferences on the causality of the results; a longitudinal approach may be beneficial in future research.

### Implications

The results of this study show that adolescents with lower SES and lack of schoolwork support are more likely to feel indifferent toward school and education. These findings imply that focusing on less socioeconomically privileged adolescents, particularly boys who experience a disrupted social context and learning difficulties (Bosakova et al., 2020), and providing them with sources of help with schoolwork may improve their attitudes toward school and education and mitigate the impact of their disadvantages.

Furthermore, we found that the indifference toward school and education among adolescents with low and middle SES decreased with increasing sources of schoolwork support inside and outside of the family. This implies that creating a stimulating environment for less privileged adolescents at school, i.e., adapting designated areas at school and spending time with trained professionals during reserved time while focusing on the experienced shortcomings, may help adolescents improve their results and satisfaction (Allington et al., 2010; Salisu and Ransom, 2014). Moreover, creating a stimulating environment at school may substitute the support absent at home for disadvantaged adolescents due to their family socioeconomic situation (García and Weiss, 2018). Additionally, even though support with schoolwork from parents may lack among the adolescents from low-SES families, appropriate interventions aimed at increasing parental need-supportive practices may reduce homework stress experienced by children (Moé et al., 2020) and providing adolescents with structured homework environment and encouraging their autonomy and efficacy at home may help with their further academic outcomes (Daw, 2012; O’Sullivan et al., 2014).

Our findings on the associations of low family SES and lack of schoolwork support with school satisfaction require confirmation in a longitudinal study to assess causal relationships. The findings show modification of schoolwork support on the associations of low family SES and school satisfaction, which may be complemented by identifying other possible ways of overcoming social inequalities of adolescents and improving their attitudes toward school and education using targeted interviews or focus groups with vulnerable groups or individuals. Moreover, the exploration of schoolwork support may be broadened by measuring the extent of support rather than the sources alone. Additionally, the results of this study may only be generalized on countries with similar educational systems. Further research is needed to confirm our findings in countries that employ institutionalized support and tutoring for the disadvantaged children within their educational system.

## Conclusion

Adolescents with lower levels of SES who do not have any source of support with schoolwork are more likely to feel indifferent with regards to school and education. Schoolwork support provided by family members or other competent adults may improve adolescents’ school satisfaction while helping them overcome the disadvantages resulting from their family SES.

## Data Availability Statement

The raw data supporting the conclusions of this article will be made available by the authors, without undue reservation.

## Ethics Statement

The studies involving human participants were reviewed and approved by the Ethics Committee of the Medical Faculty at P.J. Šafárik University in Košice (16N/2017). Written informed consent to participate in this study was provided by the participants’ legal guardian/next of kin.

## Author Contributions

SH participated in design of the study and coordination, drafted the manuscript, participated in the analyses, and interpreted the data. DH and AM participated in the design and coordination of the study, interpreted the data, helped to draft the manuscript, and provided supervision. AW and SR participated in the interpretation of the data, contributed with their comments to the final version, and provided supervision. All authors read and approved the final manuscript.

## Conflict of Interest

The authors declare that the research was conducted in the absence of any commercial or financial relationships that could be construed as a potential conflict of interest.

## Publisher’s Note

All claims expressed in this article are solely those of the authors and do not necessarily represent those of their affiliated organizations, or those of the publisher, the editors and the reviewers. Any product that may be evaluated in this article, or claim that may be made by its manufacturer, is not guaranteed or endorsed by the publisher.
